# Elderhood as an Anti-Ageism Intervention

**DOI:** 10.1093/geroni/igab046.1920

**Published:** 2021-12-17

**Authors:** Tracey Gendron, Shannon Arnette, Jenny Inker, Sarah Marrs, Maddie McIntyre, Waters Bert

**Affiliations:** Virginia Commonwealth University, Richmond, Virginia, United States

## Abstract

Ageism is a complex, multi-layered phenomenon impacting feelings, thoughts and behaviors toward self and others. Due to the complexity of ageism, evidence-based anti-ageism interventions have proved challenging and costly. To date, using the concept of elderhood as a mechanism to mitigate the negative impacts of ageism has not been explored. As an anti-ageism strategy, elderhood reframes later life as a stage that encompasses growth and development and expected loss and decline. The current study evaluated a brief video intervention among first-year medical students before participating in a year-long senior mentoring program. First-year medical students (N = 585) from 2018-2021 responded to open-ended questions after viewing the video. Thematic analysis revelated four themes: neutrality, elderhood as development, reframing stigma and elderhood as othering. Findings suggest that elderhood may be a viable and productive anti-ageism strategy.

